# The association between physical activity and subjective well-being among adolescents in southwest China by parental absence: a moderated mediation model

**DOI:** 10.1186/s12888-023-04982-8

**Published:** 2023-07-10

**Authors:** Ming Zhang, Xiaohe Xu, Jianjun Jiang, Yuanyi Ji, Ruixi Yang, Qijiao Liu, Shiying Li, Yuchen Li, Qiaolan Liu

**Affiliations:** 1 Department of Health Behavior and Social Medicine, West China School of Public Health and West, Research Center for Palliative Care, China Fourth Hospital, West China-PUMC C.C. Chen Institute of Health, Sichuan University, Chengdu, P.R. China; 2 Department of Sociology, University of Texas at San Antonio, San Antonio, USA; 3 Department of Sociology and Psychology, School of Public Administration, Sichuan University, Chengdu, P.R. China; 4 Department of Palliative Care, West China School of Public Health and West, Research Center for Palliative Care, China Fourth Hospital, West China-PUMC C.C. Chen Institute of Health, Sichuan University, Chengdu, P.R. China; 5 Nosocomial Infection Management Department, West China School of Public Health and West China Fourth Hospital, Sichuan University, Sichuan Chengdu, P.R. China; 6 Mental Health Center, West China Hospital of Sichuan University, Chengdu, P.R. China

**Keywords:** Adolescent, Parental absence, Physical activity, Subjective well-being, School connectedness, Resilience

## Abstract

**Objectives:**

Built on the *Positive Youth Development* (PYD) framework, this study examined how physical activity affected the subjective well-being of adolescents in the multi-ethnic area of southwest China. The mediating role of school connectedness as an external development asset and the moderating role of resilience as an internal development asset were specified and tested within the framework of sport-based PYD.

**Methods:**

A cross-sectional survey of 3143 adolescents (47.2% boys with mean age = 12.88 and *SD* = 1.68) was conducted in 2020. A structural equation model (SEM) was developed to estimate the direct effect of physical activity, the mediating effect of school connectedness, and the moderating effect of resilience on adolescents’ subjective well-being. Multi-group comparison was made to investigate differences and similarities across three parental absence subgroups: (1) both parents present, (2) one parent absent, and (3) both parents absent.

**Results:**

As surmised, physical activity, school connectedness, and resilience all positively and significantly affected adolescents’ subjective well-being. SEM analyses revealed that school connectedness mediated the effect of physical activity on subjective well-being. Moreover, resilience moderated both the direct and indirect effects of physical activity (through school connectedness) on subjective well-being. Finally, the multi-group comparison revealed a moderating effect of parental absence on the moderated mediation model.

**Limitations:**

This study is a cross-sectional survey, so inference of causal associations among the study variables is impossible.

**Conclusions:**

Healthy lifestyle behaviors, school-supportive settings, and positive individual development assets can enhance the subjective well-being of adolescents in southwest China, especially those whose parents were absent. Physical activity interventions informed by the PYD framework should be incorporated into public health programs designed to foster the physical and mental health of left-behind adolescents in southwest China.

**Supplementary Information:**

The online version contains supplementary material available at 10.1186/s12888-023-04982-8.

## Introduction

In the areas of adolescent mental health and psychological well-being, parental absence has been recognized as a serious stressful life event that often leads to negative emotions and deleterious mental health outcomes, such as anxiety, depression, and suicidal ideation. Such a stressful life event is also linked to mental illness in adulthood [1–4]. In the wake of the global economy, many low and middle-income countries, including China, have witnessed large-scale labor migration from rural regions to urban centers. Coupled with increased divorce rates, labor migration has given rise to many left-behind adolescents vulnerable to behavioral and mental health risks [2, 5]. The detrimental effects of parental absence on the physical and mental health of left-behind adolescents have received widespread attention from scholars and policymakers.

Since the economic reforms that began in the late 1970s, China has experienced unprecedented socioeconomic growth. In search of better job opportunities and livelihood, hundreds of millions of rural residents embarked on a journey to migrate from underdeveloped central and western provinces to developed south-eastern urban provinces. However, due to the limited access to housing, education, and healthcare in cities, most migrants chose to leave their children behind under the care of their spouses, parents, or relatives. The number of left-behind children in rural China exceeded 61 million in 2013, accounting for 21.88% of children in China [6]. The left-behind children were predominantly concentrated in large labor export provinces such as Sichuan, Henan, and Anhui [6]. As such, parental absence has become the norm for adolescents in less-developed rural China, and this is more serious in the southwest of China. Therefore, it is important to improve the mental health of adolescents with parental absence in this region. Based on the background of a large number of left-behind children in southwest China, in this study, we divided the adolescents' left-behind situation into three categories: (1) both parents present, (2) one parent absent (i.e., one parent went out to work and the other left behind), and (3) both parents absent (i.e., both parents went out to work). It is in this broader context that we examine how protective factors such as physical activity and development assets such as school connectedness and resilience work in tangent to improve adolescents’ subjective well-being during the critical period of growth (i.e., adolescence). Such endeavor can inform evidence-based prevention programs to meet the healthcare needs of adolescents, especially those whose parents are absent in less-developed and multi-ethnic southwest China.

### Physical activity and subjective well-being

Over the past several decades, most of the extant studies have used deficit models to examine the effects of parental absence on adolescents' mental health problems or poor psychological outcomes (e.g., depression and low self-esteem) and risk-taking behaviors (e.g., drinking and smoking) during adolescence [7–9]. Few studies have explored the effects of parental absence on adolescents’ subjective well-being using the positive psychology perspective, which focuses on how enhanced external development assets (such as social and school support) and internal development assets (such as resilience and hope) can improve the subjective well-being of adolescents whose parents are absent [10]. Under this positive psychology framework, subject well-being is defined as the cognitive and affective assessment of life, encompassing perceptions of life satisfaction and reactions to both positive and negative emotions [11, 12]. Previous studies have shown that improved subjective well-being for adolescents is significantly associated with reduced depressive symptoms and behavioral problems, increased academic achievement, enhanced psychosocial functions, improved interpersonal relationships, and lowered depression in adulthood [13–15]. In other words, bolstering subjective well-being can yield both short- and long-term beneficial effects on adolescents’ developmental outcomes.

Engaging in active and planned physical activity is a hallmark of healthy lifestyle behavior. According to the 2018 *Physical Activity Guidelines Advisory Committee Scientific Report*, physical activity can improve sleep quality and cognitive function and reduce the risk of depression and anxiety in adolescents [16]. The *Guidelines on Physical Activity and Sedentary Behavior* issued by the World Health Organization (WHO) in 2020 recommended that adolescents should engage in moderate to vigorous physical activity for an average of 60 min (1 h) per day [17]. However, with the transition from childhood to adolescence, adolescents will gradually experience increased academic stress, more sedentary behavior, and decreased physical activity [18, 19]. For example, the rate of physical inactivity among global school-aged adolescents is about 80%, and the proportion of adolescents with physical inactivity has gradually increased worldwide [20]. Though physical activity has been shown to reduce mental illness and enhance psychological well-being for adolescents [18], more systematic evidence is needed to further confirm the association between participation in physical activity and subjective well-being from the perspective of positive psychology [21].

Over the past decade or so, some researchers have proposed a sport-based framework for *Positive Youth Development* (PYD). The core tenet of this framework is that participation in sports can help students acquire and develop psychosocial skills and internal development assets [22]. Moreover, life skills acquired through sports experiences can be further transmitted to life experiences. As such, sports experiences formed by the interplay of individual, relational, and environmental factors can positively contribute to the generation and transmission of individual assets in adolescents, thus positively affecting their long-term development [23]. This theoretical framework can be extended to the general population of adolescents. In terms of public health practice, the PYD framework can inform the intervention strategies and programs designed to improve adolescents’ psychological functioning and mental health by substantiating the association between participation in organized physical activity and subjective well-being for adolescents.

### The mediating role of school connectedness

As an external development asset, school connectedness can be defined as the extent to which students feel accepted, respected, included, and supported by peers in the school environment, as exemplified by teachers’ attention to students’ academic and personality development and frequent interactions with peers [24, 25]. As school-aged adolescents spend most of their time in school settings, their experiences at school can have profound impacts on their physical and mental health. Stated differently, school connectedness is a key factor that promotes positive youth development [26, 27]. Some past studies have shown that lower levels of school connectedness are associated with depression, anxiety, lower levels of well-being in adolescence, and higher levels of depression in adulthood [28, 29]. On the other hand, higher levels of school connectedness are associated with higher levels of well-being, reduced risky behaviors such as suicidal attempts in adolescence, and lower incidence of health risky behaviors in adulthood [24, 30–32]. The beneficial effects of active participation in physical activity and the formation of strong school connectedness on positive psychological outcomes have been widely documented in previous studies [18, 33]. As physical activity can enhance the psychological well-being of adolescents by improving essential sociopsychologicals and life skills and facilitating social integration [34], it is hypothesized that active participation in physical activity will enhance the subjective well-being of adolescents through improved school connectedness. That is, school connectedness will mediate the positive association between participation in physical activity and subjective well-being among adolescents in less developed and multi-ethnic southwest China.

### The moderating role of resilience

Resilience is commonly recognized as an internal development asset that promotes positive youth development. Under the PYD framework, resilience is conceptualized as the adaptive and interactive relationship between adolescents and the environment in which they live. More specifically, resilience refers to adolescents’ ability to adapt to disruptions in growth triggered by risk or adversity [35, 36]. Several studies conducted in China have revealed that a high level of resilience among adolescents is strongly associated with a heightened sense of school-based support and a high level of subjective well-being [37, 38]. As such, resilience is often perceived as a protective factor or a buffer for adolescents’ mental health. Thus far, scholars have proposed three models of resilience based on different action mechanisms in coping with different environments: (1) the protective-reactive model in which resilience is conceptualized to buffer the adverse effects of stressors and reduce the occurrence of negative outcomes; (2) the compensatory model that utilizes stress factors and resilience to predict the development of individuals; and (3) the challenge model under which different levels of stress are said to have different effects on the development of individuals; that is, while low levels of stress can promote the positive development, high levels of stress can generate harmful effects on individuals [39]. In addition, some researchers have proposed the protective-protective model, in which resilience as a protective factor can enhance the impact of another protective factor on developmental outcomes [40]. Therefore, the two patterns of the protective model exemplify the different roles of resilience as moderators, namely, buffering the effects of risk factors or promoting the effects of protective factors.

Indeed, previous studies have often used resilience as a buffer that moderated the negative effects of risk or adversity (e.g., bullying and trauma) on subjective well-being in adolescents [41–43]. Few studies have examined the positive impact of resilience in promoting the effects of other protective factors on subjective well-being from a protective-protective model. Although a Chinese study demonstrated that resilience amplified the positive effects of peer caring on adolescents' subjective well-being [44], more empirical evidence is needed to substantiate the moderating role of resilience from a positive psychology perspective. To fulfil this research goal, the present study tests the hypothesis that adolescents’ resilience will moderate the direct and indirect pathways through which physical activity will positively affect school connectedness, which in turn will positively affect subjective well-being among adolescents in southwest China. That is, the higher the level of resilience, the stronger the positive effects of physical activity will have on school connectedness and consequently subjective well-being.

## The present study

**Fig. 1:**
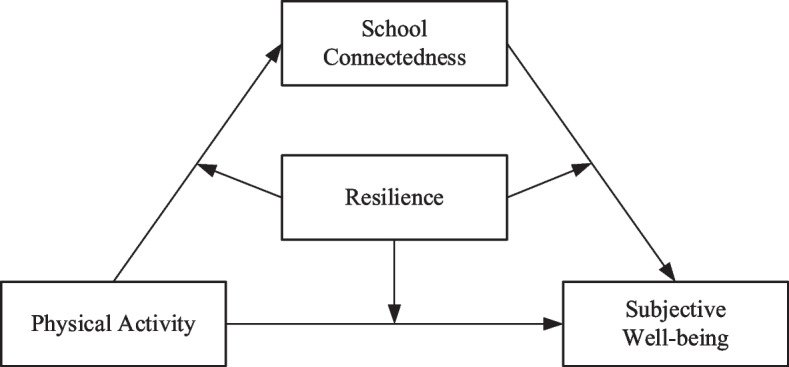
Hypothetical model of the associations among physical activity, school connectedness, resilience, and subjective well-being among adolescents in southwest China

To summarize, informed by the PYD framework in general and the sport-based PYD perspective in particular, this study tests the following four hypotheses that are depicted in Fig. 1. **H1:** Active participation in physical activity is hypothesized to be positively associated with the subjective well-being of adolescents in southwest China. **H2:** School connectedness as an indicator of external development assets will mediate the positive association between physical activity and subjective well-being. **H3:** Resilience as an indicator of internal development assets will moderate both direct and indirect pathways from physical activity to subjective well-being through school connectedness. **H4:** The moderated mediation model as hypothesized in H2 and H3 will be different across the three parental absence subgroups, namely, both parents were present, one parent was absent, and both parents were absent.

## Methods

### Participants and procedure

A cross-sectional survey was conducted from April to October 2020 to explore the patterns of health education for adolescents in ethnic minority agglomerations in southwest China. Based on the characteristics and residential patterns of each ethnic minority group, one elementary school and one junior high school were selected from three locales: (1) Cangxi County, Guangyuan City of Sichuan province, (2) Ganluo County, the Liangshan Yi Autonomous Prefecture of Sichuan province, and (3) Lhasa City of the Tibet Autonomous Region. All elementary school students in grades 5 and 6 and junior high school students were included in the survey that featured the self-administered questionnaire. The ethical approval of the data collection was obtained from the Medical Ethics Committee of Sichuan University (No. K2018086).

All investigators in this study were researchers and graduate students in public health and psychology-related disciplines. Before the survey was fielded, all investigators were trained by experienced researchers. Informed consent was secured before the survey was conducted. During the survey, the investigators were required to provide information about the purpose and content of the survey to the participants. The survey took place in the same period of time at each school, and the participants were required to fill out the questionnaire independently. At the end of the survey, the investigators performed data entry and data cleaning.

A total of 3195 adolescents participated in the survey. After removing cases with incomplete records (i.e., samples with more than 20% of items not answered, especially those related to the study variables), a subsample of 3143 adolescents was included in the present study. Of the 3143 adolescents, 1483 (47.2%) were male and 1660 (52.8%) were female with an average age of 12.88 ± 1.68 years. As indicated previously, the survey captured three major ethnic groups in southwest China: Yi (836; 26.6%), Tibetan (701; 22.3%), and Han (1606; 51.1%). In terms of parental absence, 1889 (60.1%) adolescents reported that both parents were present, 729 (23.2%) reported that one parent was absent, and 525 (16.7%) reported that both parents were absent.

### Measures

#### Parental absence

One questionnaire item, “Are your parents absent (not around to take care of you)?”, was used to determine parental absence with three response categories: 1 = “both parents present”, 2 = “one parent absent”, and 3 = “both parents absent”.

#### Subjective well-being

Subjective well-being is a latent endogenous variable, which was measured by the World Health Organization Well-Being Index (WHO-5). This index contained five items that evaluated positive aspects of mental health [45]: (1) “I have felt cheerful and in good spirits”, (2) “I have felt calm and relaxed”, (3) “I have felt active and vigorous”, (4) “I woke up feeling fresh and rested”, and (5) “My daily life has been filled with things that interest me”. Responses to these items were scored on a 6-point scale representing 1 = “at no time”, 2 = “some of the time”, 3 = “less than half the time”, 4 = “more than half the time”, 5 = “most of the time”, and 6 = “all of the time”. The scores of the five items were summed with higher scores indicating better subjective well-being. The index demonstrated good psychometric properties such as internal consistency and validity in the Chinese context [46]. In this study, Cronbach’s alpha coefficient for the subjective well-being index was 0.94, and McDonald’s omega coefficient was 0.94.

#### Physical activity

Physical activity is a latent exogenous variable. It was measured by duration, intensity, and frequency [47]. First, the average time of physical activity per day was a 4-point scale with 1 = “none”, 2 = “ < 30 min”, 3 = “30 ~ 60 min”, and 4 = “ > 60 min”. Second, the number of days that physical activity exceeded 30 min in the past week was a 5-point scale with 1 = “0 days”, 2 = “1 ~ 2 days”, 3 = “3 ~ 4 days”, 4 = “5 ~ 6 days”, and 5 = “7 days”. Third, the frequency of participating in physical activity in the past 30 days was a 4-point scale with 1 = “6 ~ 7 times per week”, 2 = “3 ~ 5 times per week”, 3 = “1 ~ 2 times per week”, and 4 = “I didn't participate in physical activity”. Finally, the cumulative time spent per physical activity in the past 30 days was a 3-point scale with 1 = “I didn't participate in physical activity”, 2 = “ < 30 min”, and 3 = “ ≥ 30 min”. The third item was reverse-coded. The scores of the four items were summed, and higher scores represented more frequent physical activities. Adolescents who participated in physical activity ≥ 3 times per week and spent a cumulative time ≥ 30 min per physical activity in the past 30 days achieved the standard of physical activity. The results of the confirmatory factor analysis (CFA) were as follows:$\chi^{2}$/*df* = 2.079, AGFI = 0.997, RFI = 0.995, TLI = 0.998, SRMR = 0.005, and RMSEA = 0.019, indicating a good fit. Cronbach’s alpha coefficient of the physical activity composite variable was 0.72, and McDonald’s omega coefficient was 0.74. The above results indicated that the reliability and validity of the scale were good.

#### School connectedness

School connectedness is a latent mediating variable. It came from the National Longitudinal Study of Adolescent Health (Add Health) [48]. The scale was comprised of 5 items: (1) “I feel close to people at this school”, (2) “I am happy to be at this school”, (3) “I feel like I am part of this school”, (4) “The teachers at this school treat students fairly”, and (5) “I feel safe in my school”. Responses were scored on a 5-point Likert scale with 1 = “strongly disagree”, 2 = “disagree”, 3 = “neither disagree nor agree”, 4 = “agree”, and 5 = “strongly agree”. The five items were summed with higher scores representing stronger school connectedness. Cronbach’s alpha coefficient and McDonald’s omega coefficient for this scale were respectively 0.85 and 0.86, which was consistent with other studies conducted in China [49].

#### Resilience

Resilience is a latent moderating variable. It was measured by the Connor-Davidson Resilience Scale (CD-RISC) developed by Connor and Davidson in 2003 [50]. The scale was comprised of 25 items, such as “I'm able to adapt to change”, “I have close and secure relationships”, and “I can achieve my goals” (see Appendix 2 for details). All responses were scored on a 5-point scale ranging from 0 to 4 with 0 = “not true at all”, 1 = “rarely true”, 2 = “sometimes true”, 3 = “often true”, and 4 = “true nearly all of the time”. This scale was further decomposed into three subscales of tenacity, strength, and optimism [51]. Both the overall scale and subscales achieved good reliability and validity in evaluating the resilience of Chinese adolescents [52]. In this study, Cronbach's alpha coefficients for the three subscales of tenacity, strength, and optimism were 0.96, 0.91, and 0.83, respectively. And McDonald’s omega coefficients of the three subscales were 0.96, 0.91, and 0.84, respectively.

#### Control variables

In this study, we investigated the demographic characteristics of adolescents such as gender, age, and ethnicity, as well as family background information such as family economic status and parental education level. Demographic characteristics of adolescents, such as gender, age, and ethnicity, were statistically controlled for as confounding factors in the subsequent statistical analyses. While age was used as a continuous variable, gender and ethnic identify were dummy-coded.

### Data analysis

To test the study hypotheses, descriptive, bivariate, and multivariate statistical analyses were performed. First, the descriptive analysis was carried out to report the mean, standard deviation (denoted as *mean* ± *standard deviation*), or percentage distribution (denoted as *n* (%)) for each of the study variables. Second, one-way analysis of variance (ANOVA) was used to compare differences in subjective well-being, physical activity, school connectedness, and resilience across the three parental absence subgroups. Third, to adjust for gender, age, and ethnicity, the partial correlation analysis was conducted for subjective well-being, physical activity, school connectedness, and resilience. Fourth, a structural equation model (SEM) with latent variables was developed to examine the effects of adolescents’ participation in physical activity on their subjective well-being. The unconstrained approach was taken to estimate the moderating effects of the latent variables denoted by the indicators of interaction terms [53, 54]. While evaluating the goodness-of-fit, the following statistics were reported: the goodness-of-fit index (GFI > 0.90), the adjusted goodness-of-fit index (AGFI > 0.90), the normed fit index (NFI > 0.90), the relative fit index (RFI > 0.90), the incremental fit index (IFI > 0.90), the Tucker-Lewis index (TLI≈0.95), the comparative fit index (CFI≈0.95), and the root mean square error of approximation (RMSEA < 0.05) [55]. To test the moderated (i.e., by resilience) mediation model, this study made use of the *bias-corrected percentile method* with 5000 bootstrap samples. Last but not least, a multi-group comparison was made to explore differences and similarities in the moderated mediation model across the parental absence subgroups. All statistical analyses were conducted using SPSS version 24.0 (IBM, Armonk, NY, USA) and AMOS version 24.0 (IBM, Armonk, NY, USA). The significance level for all analyses was defined as *P* ≤ 0.05 (two-tailed).

## Results

### Common method bias

The survey in this study was conducted using a self-administered questionnaire for adolescents, which may lead to common method bias. To reduce the effects of it, for procedural remedies, the questionnaire used in this study included reverse-coded items and we emphasized to the participants that there were no standard answers to items. For statistical remedies, controlling for the effects of a single unmeasured latent method factor (ULMC) was used to test for common method bias [56]. We added a method factor to the model, with all items as indicators of the factor. If the changes in the fit indices of the new model aren't significant compared to the original model (e.g., the increase of TLI and CFI doesn't exceed 0.1 and the decrease of RMSEA doesn't exceed 0.05), it indicates that there is no serious common method bias [57]. In this study, the addition of the method factor resulted in non-significant changes in the fit indices of the model (ΔTLI = 0.031, ΔCFI = 0.032, and ΔRMSEA = 0.012), indicating the absence of serious common method bias.

### Preliminary analysis

Table 1 reports descriptive statistics for the variables under this study by parental absence subgroups. As can be observed from the table, except for gender, age (*P* < 0.01), ethnicity (*P* < 0.001), family economic status (*P* < 0.001), as well as father's (*P* < 0.001) and mother's (*P* < 0.001) education levels differed significantly across the three parental absence subgroups. In terms of age, adolescents with one parent absent were on average significantly younger than those whose both parents were either present or absent. Turning to ethnic identity, Yi and Han adolescents were more likely to report absent parents than their Tibetan peers. In terms of family economic status, adolescents with both parents present were better off than those with at least one parent absent. In terms of parental education level, among adolescents with both parents present, the proportion of adolescents whose parents have a junior college or better education was significantly higher than those with one parent absent and both parents absent. Moreover, the subjective well-being of adolescents with one parent absent was significantly and unexpectedly higher than those whose both parents were present or absent (*P* < 0.01). Physical activity was significantly lower among adolescents with both parents present than those with at least one parent absent (*P* < 0.01). Furthermore, adolescents with both parents absent had significantly lower resilience than adolescents with both parents present or one parent absent (*P* < 0.01). However, there was no significant difference in school connectedness among adolescents across the three parental absence subgroups.

**Table 1:** One-way ANOVA for adolescents with different parental absence

**Variable**	**Both parents present(** ***n*** ** = 1889)**	**One parent absent(** ***n*** ** = 729)**	**Both parents absent(** ***n*** ** = 525)**	***F*** **/** $\chi^{2}$	***P***
	**(** ***M*** ** ± ** ***SD*** **)/** ***n*** **(%)**	**(** ***M*** ** ± ** ***SD*** **)/** ***n*** **(%)**	**(** ***M*** ** ± ** ***SD*** **)/** ***n*** **(%)**		
**Age**	12.944 ± 1.732 _a_	12.676 ± 1.630 _b_	12.949 ± 1.533 _a_	7.188	0.001
**Gender**				0.376	0.829
Male	883(46.7) _a_	348(47.7) _a_	252(48.0) _a_		
Female	1006(53.3) _a_	381(52.3) _a_	273(52.0) _a_		
**Ethnicity**				380.981	< 0.001
Yi	564(29.9) _a_	142(19.5) _b_	130(24.8) _a, b_		
Han	720(38.1) _a_	520(71.3) _b_	366(69.7) _b_		
Tibetan	605(32.0) _a_	67(9.2) _b_	29(5.5) _c_		
**Family economic status**				47.110	< 0.001
Wealthy	357(18.9) _a_	77(10.6) _b_	52(9.9) _b_		
General	1168(61.8) _a_	517(70.9) _b_	378(72.0) _b_		
Poor	364(19.3) _a_	135(18.5) _a_	95(18.1) _a_		
**Father's education level**				114.879	< 0.001
Illiterate or semi-literate	233(12.3) _a_	54(7.4) _b_	34(6.5) _b_		
Elementary school	360(19.0) _a_	162(22.2) _a_	116(22.1) _a_		
Junior high school	415(22.0) _a_	192(26.4) _b_	185(35.2) _c_		
High school or technical secondary school	307(16.3) _a_	151(20.7) _b_	94(17.9) _a, b_		
Junior college and above	238(12.6) _a_	51(7.0) _b_	11(2.1) _c_		
Unknown	336(17.8) _a_	119(16.3) _a_	85(16.2) _a_		
**Mothers' education level**				119.481	< 0.001
Illiterate or semi-literate	380(20.1) _a_	77(10.6) _b_	76(14.5) _c_		
Elementary school	372(19.7) _a_	196(26.9) _b_	124(23.6) _b_		
Junior high school	340(18.0) _a_	184(25.2) _b_	153(29.2) _b_		
High school or technical secondary school	259(13.7) _a_	105(14.4) _a_	71(13.5) _a_		
Junior college and above	188(10.0) _a_	38(5.2) _b_	8(1.5) _c_		
Unknown	350(18.5) _a_	129(17.7) _a_	93(17.7) _a_		
**Physical activity**				28.947	< 0.001
Achievement of standard	461(24.4) _a_	235(32.2) _b_	180(34.3) _b_		
Below standard	1428(75.6) _a_	494(67.8) _b_	345(65.7) _b_		
**School connectedness**	18.911 ± 4.259 _a_	19.019 ± 3.922 _a_	18.705 ± 3.882 _a_	0.900	0.407
**Subjective well-being**	18.901 ± 7.226 _a_	19.620 ± 6.910 _b_	18.215 ± 6.889 _a_	6.105	0.002
**Overall resilience**	57.117 ± 24.968 _a_	57.464 ± 23.794 _a_	53.398 ± 22.463 _b_	5.446	0.004
Tenacity^a^	29.874 ± 13.547 _a_	29.642 ± 13.303 _a_	27.276 ± 12.381 _b_	8.009	< 0.001
Strength^a^	18.539 ± 8.211 _a, b_	19.005 ± 7.667 _a_	17.890 ± 7.300 _b_	3.014	0.049
Optimism^a^	8.704 ± 4.263 _a_	8.816 ± 4.014 _a_	8.232 ± 4.034 _b_	3.373	0.034

^**a**^ Tenacity, strength, and optimism are the three subscales of resilience

_**a, b, c**_Each subscripted letter represents a category subset of parental absence, and there are significant differences among the values of columns with different subscripted letters at the *α* = 0.05 test level

Table 2 displays the partial correlation coefficients adjusting for gender, age, and ethnicity. As anticipated, net of sociodemographic controls, all study variables were significantly and positively correlated. That is, physical activity was positively correlated with school connectedness, subjective well-being, and resilience. School connectedness was also positively correlated with subjective well-being and resilience. Additionally, resilience was positively correlated with subjective well-being.

**Table 2:** Descriptive statistics and partial correlation analysis among variables (*N* = 3143)

Variable	*M* ± *SD*	1	2	3	4	5	6	7
1. Physical activity	9.948 ± 2.479	1						
2. School connectedness	18.901 ± 4.122	0.174^***^	1					
3. Subjective well-being	18.953 ± 7.110	0.247^***^	0.347^***^	1				
4. Resilience	56.576 ± 24.331	0.249^***^	0.244^***^	0.467^***^	1			
5. Tenacity	29.386 ± 13.332	0.232^***^	0.237^***^	0.449^***^	0.974^***^	1		
6. Strength	18.539 ± 7.946	0.255^***^	0.240^***^	0.460^***^	0.958^***^	0.887^***^	1	
7. Optimism	8.651 ± 4.172	0.219^***^	0.205^***^	0.405^***^	0.871^***^	0.775^***^	0.832^***^	1

^***^P < 0.001 (two-tailed), adjusting for gender, age and ethnicity

### Testing of moderated mediation model

The multivariate statistical analysis began with a multicollinearity test, which generated a series of variance inflation factors (VIF) that are less than 2, indicating that no multicollinearity problem surfaced (results are shown in Appendix Table 1 of Appendix 1). Given these results, a structural equation model with latent variables was constructed to test the mediating role of school connectedness through which physical activity is linked to subjective well-being net of statistical controls. The goodness-of-fit indices for the mediation model were $\chi^{2}$/*df* = 9.005, GFI = 0.961, AGFI = 0.949, NFI = 0.957, RFI = 0.950, IFI = 0.962, TLI = 0.955, CFI = 0.962, and RMSEA = 0.050, which collectively indicated a good fitting model. The path coefficients showed that all three paths, namely, from physical activity to school connectedness, from physical activity to subjective well-being, and from school connectedness to subjective well-being, were statistically significant. Physical activity was significantly and positively associated with school connectedness (*β* = 0.246, *t* = 10.634, *P* < 0.001) and subjective well-being (*β* = 0.209, *t* = 10.174, *P* < 0.001), whereas school connectedness was significantly and positively associated with subjective well-being (*β* = 0.339, *t* = 16.753, *P* < 0.001) (the mediation model and the significance of path coefficients are shown in Appendix Fig. 1 and Appendix Table 2 of Appendix 1). Moreover, the bootstrap method with 5000 samples was used to generate confidence intervals for the path coefficients. The results are displayed in Table 3. As shown in the table, the bootstrap 95% confidence intervals for the direct and total effect values of physical activity on subjective well-being and the indirect (mediation) effect value of school connectedness didn’t contain 0, indicating that school connectedness mediated the pathway from physical activity to subjective well-being. The mediation effect value (0.083) accounted for 28.4% of the total effect value (0.292). Taken together, these results provided strong support for H1 and H2.

**Table 3:** Testing the mediating role of school connectedness

Effect / Effect ratio	Effect value	Boot *SE*	Boot *CI* lower limit	Boot *CI* upper limit	*P*
**Indirect (mediation) effect**	0.083	0.009	0.066	0.103	< 0.001
**Direct effect**	0.209	0.022	0.167	0.251	< 0.001
**Total effect**	0.292	0.021	0.251	0.333	< 0.001
**Mediation effect ratio**	0.286	0.035	0.225	0.361	< 0.001

**Fig. 2:**
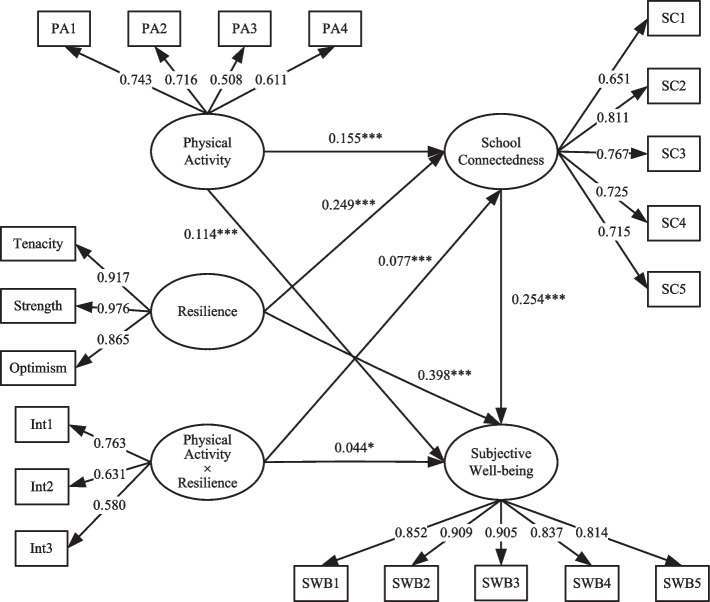
Standardized SEM coefficients (*N* = 3143) for the association between physical activity and subjective well-being among adolescents in southwest China: the mediating role of school connectedness and the moderating role of resilience **Notes: **PA1-PA4 represent the 4 items of the physical activity scale, SC1-SC5 represent the 5 items of the school connectedness scale, and SWB1-SWB5 represent the 5 items of the WHO-5 index. Tenacity, strength, and optimism represent the 3 subscales of resilience. Int1-Int3 represent the indicators of the interaction term between physical activity and resilience. This study also examined the interaction of school connectedness and resilience, which is not shown in the figure because the interaction term is not statistically significant. The path coefficients of the control variables (i.e., gender, age, and ethnicity) are omitted. ^*^P < 0.05 (two-tailed). ^***^P < 0.001 (two-tailed)

Table 4 displays the moderating role of resilience in the mediation model as shown above. The goodness-of-fit indices for the moderated mediation model were $\chi^{2}$/*df* = 5.542, GFI = 0.962, AGFI = 0.953, NFI = 0.960, RFI = 0.955, IFI = 0.967, TLI = 0.962, CFI = 0.967, and RMSEA = 0.038, indicating a good fit. A careful examination of the table revealed that resilience only moderated part of the mediation model. That is, the pathway from physical activity to school connectedness and the pathway from physical activity to subjective well-being. The interaction term for physical activity and resilience was significantly and positively associated with school connectedness (*β* = 0.077, *t* = 3.554, *P* < 0.001) and subjective well-being (*β* = 0.044, *t* = 2.320, *P* < 0.05). As such, the moderating role of resilience involved primarily physical activity, which partially moderated mediation model as displayed in Fig. 2.

**Table 4:** Path coefficients of the moderated mediation model

Path	Unstandardized coefficient	Standardized coefficient	*SE*	*t*	*P*
**Physical activity** → **School connectedness**	0.186	0.155	0.028	6.708	< 0.001
**School connectedness** → **Subjective well-being**	0.518	0.254	0.039	13.214	< 0.001
**Physical activity** → **Subjective well-being**	0.279	0.114	0.048	5.845	< 0.001
**Resilience** → **School connectedness**	0.046	0.249	0.004	11.767	< 0.001
**Resilience** → **Subjective well-being**	0.150	0.398	0.007	21.304	< 0.001
**Physical activity** $\times$ **Resilience** → **School connectedness**	0.033	0.077	0.009	3.554	< 0.001
**Physical activity** $\times$ **Resilience** → **Subjective well-being**	0.039	0.044	0.017	2.320	0.020

**Notes: **This study also examined the interaction of school connectedness and resilience, which is not shown in the table because the interaction term is not statistically significant. The path coefficients of the control variables (i.e., gender, age, and ethnicity) are omitted

To further illustrate the moderating role, resilience was divided into high (*M* + *SD*), medium (*M*), and low (*M-SD*) groups. The results are reported in Table 5. As anticipated, the positive association between physical activity and school connectedness was significant at high resilience (*β* = 0.255, *P* < 0.001); the positive association was weaker but still significant at medium resilience (*β* = 0.155, *P* < 0.001); and the association was no longer statistically significant at low resilience (*β* = 0.056, *P* > 0.05). In a similar vein, the positive association between physical activity and subjective well-being was significant at high resilience (*β* = 0.172, *P* < 0.001); the positive association was weaker but still significant at medium resilience (*β* = 0.114, *P* < 0.001); and the association was no longer statistically significant at low resilience (*β* = 0.057, *P* > 0.05). Finally and more importantly, the mediating role of school connectedness on the pathway between physical activity and subjective well-being was significant (*β* = 0.060, *P* < 0.001) at high resilience (25.9%); the mediating role was weaker but still significant (*β* = 0.040, *P* < 0.001) at medium resilience (25.7%); and the mediating role wasn’t statistically significant (*β* = 0.015, *P* > 0.05) at low resilience. In other words, the mediation effect values were significantly different across the three resilience groups, which lent partial credence to H3.

**Table 5:** Testing the moderating role of resilience

Path / Effect	Effect value	Boot *SE*	Bias-corrected 95%*CI*			Percentile 95%*CI*		
			Lower	Upper	*P*	Lower	Upper	*P*
**Physical activity** → **School connectedness**								
High resilience	0.255	0.039	0.180	0.334	< 0.001	0.177	0.333	< 0.001
Medium resilience	0.155	0.024	0.106	0.202	< 0.001	0.108	0.203	< 0.001
Low resilience	0.056	0.043	-0.026	0.145	0.188	-0.026	0.145	0.183
**Mediation effect**								
High resilience	0.060	0.011	0.040	0.086	< 0.001	0.039	0.084	< 0.001
Medium resilience	0.040	0.007	0.027	0.055	< 0.001	0.026	0.054	< 0.001
Low resilience	0.015	0.012	-0.007	0.040	0.182	-0.007	0.040	0.183
**Comparison of delta-mediation effects**								
Mediation effect at high resilience—low resilience	0.045	0.018	0.011	0.081	0.011	0.010	0.079	0.013
Mediation effect at high resilience—medium resilience	0.020	0.009	0.004	0.039	0.014	0.003	0.038	0.020
Mediation effect at medium resilience—low resilience	0.025	0.009	0.007	0.043	0.009	0.006	0.043	0.011
**Direct effect**								
High resilience	0.172	0.036	0.101	0.243	< 0.001	0.100	0.242	< 0.001
Medium resilience	0.114	0.021	0.072	0.155	< 0.001	0.072	0.155	< 0.001
Low resilience	0.057	0.036	-0.014	0.127	0.109	-0.013	0.129	0.105
**Total effect**								
High resilience	0.232	0.035	0.164	0.300	< 0.001	0.163	0.299	< 0.001
Medium resilience	0.154	0.022	0.111	0.195	< 0.001	0.111	0.195	< 0.001
Low resilience	0.072	0.038	-0.001	0.149	0.056	-0.001	0.149	0.055
**Mediation effect ratio**								
High resilience	0.259	0.065	0.159	0.416	< 0.001	0.157	0.412	< 0.001
Medium resilience	0.257	0.054	0.169	0.384	< 0.001	0.169	0.383	< 0.001
Low resilience	0.211	1.831	-0.249	1.270	0.177	-0.343	1.121	0.211

### Multi-group comparison

Multi-group comparison was conducted to examine differences and similarities across different parental absence subgroups. According to the results for the multi-group comparison of adaptation and the model invariance test, it was known that except for measurement weights model, the *P*-values of structural weights model, structural covariances model, structural residuals model, and measurement residuals model invariance test were all less than 0.05. However, the absolute values of the amount of change in the model fit indices were all less than 0.05, which indicated the moderated mediation model was simultaneously adapted to adolescents with different parental absence subgroups (results are shown in Appendix Table 3a, Appendix Table 3b, and Appendix Table 4 of Appendix 1).

As reported in Table 6, compared to adolescents with both parents present, the resilience of adolescents with one parent absent had no moderating role on the pathway from physical activity to school connectedness. Moreover, for adolescents with both parents absent, the pathway from physical activity to school connectedness wasn’t statistically significant and there was no moderating role of resilience. In effect, in all parental absence subgroups, resilience had no moderating role on the direct path from physical activity to subjective well-being. However, for adolescents with both parents present, the path coefficient from school connectedness to subjective well-being was significantly lower than those whose both parents were absent (*P* < 0.01); the path coefficient from resilience to school connectedness was significantly lower than those with one parent absent (*P* < 0.05); the path coefficient from resilience to subjective well-being was significantly higher than those whose both parents were absent (*P* < 0.01); and the path coefficient from the interaction term of physical activity and resilience to school connectedness was significantly higher than those with one parent absent (*P* < 0.05) and both parents absent (*P* < 0.01). The above results partially confirmed H4.

**Table 6:** Path coefficients and critical ratios for multi-group comparison

Path	Both parents present(*n* = 1889)^a^	One parent absent(*n* = 729)^b^	Both parents absent(*n* = 525)^c^	Critical Ratio / *P-value*		
				Subgroup I compares with Subgroup II	Subgroup I compares with Subgroup III	Subgroup II compares with Subgroup III
**Physical activity → School connectedness**	0.170^***^	0.159^***^	0.103	-0.180/0.857	-0.984/0.325	-0.726/0.468
**School connectedness → Subjective well-being**	0.224^***^	0.291^***^	0.362^***^	1.636/0.102	2.609^**^/0.009	1.028/0.304
**Physical activity → Subjective well-being**	0.114^***^	0.093^*^	0.138^**^	-0.337/0.736	0.577/0.564	0.745/0.456
**Resilience → School connectedness**	0.215^***^	0.331^***^	0.328^***^	2.249^*^/0.025	1.792/0.073	-0.162/0.871
**Resilience → Subjective well-being**	0.430^***^	0.365^***^	0.269^***^	-0.935/0.350	-2.988^**^/0.003	-1.805/0.071
**Physical activity × Resilience → School connectedness**	0.128^***^	-0.010	-0.059	-2.473^*^/0.013	-2.798^**^/0.005	-0.689/0.491
**Physical activity × Resilience → Subjective well-being**	0.044	0.019	0.087	-0.463/0.643	0.917/0.359	1.111/0.267

^**a**^^**, b, c**^ Different subscript letters represent subgroups of adolescents with different parental absence

^**a**^It represents the subgroup of adolescents with both parents present which is noted as Subgroup I

^**b**^It represents the subgroup of adolescents with one parent absent which is noted as Subgroup II

^**c**^It represents the subgroup of adolescents with both parents absent which is noted as Subgroup III

^*^P < 0.05 (two-tailed)

^**^P < 0.01 (two-tailed)

^***^P < 0.001 (two-tailed)

## Discussions

Guided by insights from the sport-based PYD framework, this study examined the association between participation in physical activity and subjective well-being among multi-ethnic adolescents in less developed and multi-ethnic southwest China. Within this context, participation in school-based or organized physical activity was found to foster adolescents’ development assets and subjective well-being. In what follows, we reiterate and highlight our noteworthy findings.

First, in support of our first hypothesis (H1), we found a positive association between participation in physical activity and subjective well-being. That is, the frequent, intensive, and sustained participation in organized physical activity significantly may increase adolescents’ subjective well-being irrespective of their age, gender and ethnicity. Put differently, regular participation in organized physical activity is significantly and positively associated with adolescents’ subjective well-being in the less developed, multi-ethnic regions in southwest China. This finding is largely in line with previous studies, which demonstrated that physical activity in general is associated with improved positive emotions and life satisfaction, and participation in organized physical activity in particular enhances subjective well-being in adolescents [18, 58]. It follows that active participation in organized physical activity in school is a viable intervention strategy that can facilitate caring for adolescents’ mental health in southwest China [19, 34].

Second, as surmised (H2), school connectedness, as a measure of external development assets, significantly mediated the association between physical activity and subjective well-being net of sociodemographic controls. This finding underscores an important mechanism for the generation and transmission of development assets. That is, participation in school-based or organized physical activity can foster adolescents’ external development assets such as school connectedness, which can in turn bolster adolescents’ subjective well-being. As suggested by the sport-based PYD framework, adolescents are most likely to exhibit positive mental health outcomes when they engage in desired activities in the appropriate environment. Physical activity in school settings through interventions such as physical education classes where adolescents are coached by physical education teachers helps establish a supportive, student-centered environment. This positive and enriched environment can empower adolescents to cultivate school connectedness that may, in turn, improve their subjective well-being [23, 35]. Simply put, receiving support via school connectedness is positively associated with healthy physical and mental development for adolescents [24, 25], which is particularly important for adolescents whose parents are absent. In this context, support from school can compensate for the lack of family involvement and support due to parental absence. The understanding, caring, and sense of belonging felt at school can reduce the psychological distress triggered by parental absence [59]. It thus comes as no surprise that active participation in physical activity may not only promote school connectedness but also improve mental health outcomes for adolescents. By moving beyond the conventional conceptualization that defines school connectedness as a development asset, we specified, tested, and verified the mediating role of school connectedness. Such endeavors contribute to the growing literature on the sport-based PYD framework.

Third, consistent with our moderating hypothesis (H3), we found that resilience as an indicator of internal development assets partially modified the effect value of school connectedness that mediated the association between physical activity and subjective well-being. This moderated mediation model lends strong credence to the protective-protective model under the sport-based PYD framework [40]. It became evident that both direct and indirect relationships between physical activity (via school connectedness) and adolescents’ subjective well-being were more pronounced at the high and medium levels of resilience than that at the low level of resilience. In fact, the direct and indirect relationships of participation in physical activity with school connectedness and subjective well-being were largely nullified (insignificant) at the low level of resilience. These nuanced moderating roles involving cultivating resilience as an internal development asset provide support for previous studies that showed buffering effects against risk factors and augmenting protective factors for mental health [43, 44]. Therefore, promoting the development of internal asset such as resilience in adolescents through physical activity is also a feasible strategy to facilitate healthy growth of adolescents.

The results of the multi-group analysis revealed that the moderated mediation model constructed in this study operated in slightly different ways across different parental absence subgroups. In our study, adolescents with both parents present reported lower physical activity than those with one parent or both parents absent, and adolescents with one parent absent reported higher subjective well-being than those with both parents present or absent. The possible reason is that compared to adolescents with both parents present, adolescents with parents absent are primarily cared for by their grandparents, relatives, etc. However, due to the caregiver’s limited time, energy, and other resources, adolescents are likely to engage in physical activity as a recreational activity to enrich their lives and regulate their emotions. On the other hand, most of the adolescents in this study with one parent absent were cared for by their mothers, who therefore received more compensatory care from their mothers and were able to give them stronger psychological support, which in turn generated higher subjective well-being. The results of previous studies suggesting that adolescents with absent mothers exhibit lower levels of security and worse psychological adjustment, which have persistent negative effects on their development, can partially explain the results that emerged in the present study [60, 61]. Moreover, our analysis revealed that the positive association between school connectedness and subjective well-being was significantly higher for adolescents with both parents absent than for those with both parents present. This can be explained by the fact that the growth and development of left-behind adolescents are more likely to depend on the school environment than those with parents present. As such, their interactions with and influences from teachers and peers at school are even more prominent than those from their parents. Additionally, the moderating role of resilience was significant for adolescents whose parents were present but insignificant for adolescents with absent parents. Adolescents with absent parents may be disproportionately exposed to multiple risk factors than adolescents with both parents present, thus weakening the positive effects of resilience on school connectedness and subjective well-being [62]. However, among left-behind adolescents with both parents absent, the path coefficients of physical activity and school connectedness were statistically higher than those with both parents present and one parent absent while predicting subjective well-being. This may suggest that to improve the subjective well-being of left-behind adolescents, it is essential to implement intervention programs to simultaneously increase adolescents’ participation in organized physical activity and school connectedness that can further enhance resilience, thus alleviating the deleterious effects of cumulative and multiple risks for left-behind adolescents.

Consonant with the PYD framework, this study can yield important public health implications for designing school-based physical activity interventions. First, providing resources for adolescents to engage in sports and social activities and offering ample sports venues and fitness equipment near the school is imperative. Research has shown that adolescents with resources such as facilities and places for physical activity have exhibited improved subjective well-being through partaking in organized sports and activities [63]. Second, through daily physical education at school, the tasks and goals of the curriculum can be gradually shifted from teacher-centered to student-centered, which can strengthen students' interactions with peers and adult instructors, thus consolidating a supportive school climate and fostering adolescents’ school connectedness. Third, in addition to physical education classes, schools must capitalize on resources to organize a variety of sports-related activities, such as morning jogs, physical activity recesses, ball games, and fun games, as well as additional school-based club organizations such as track and field teams, basketball teams, and soccer teams, in order to satisfy the needs of adolescents for all-round physical activity. In so doing, adolescents can fully exploit the positive relationships of participation in physical activity with resilience and subjective well-being.

## Limitations

Although this study has both theoretical and practical implications, there are several limitations. First and foremost, this study utilized a cross-sectional survey, which would make it difficult to conduct statistical analyses to establish a causal association between participation in physical activity and subjective well-being. To provide more direct and stronger support for the sport-based PYD framework and intervention programs, longitudinal designs or experimental designs with random trails are suggested for future research. Second, the sample size among parental absence subgroups wasn’t balanced such that the findings for the subgroups with smaller sample size may not be accurate. Third, when investigating parental absence, our study used the variable with three categories: both parents present, one parent absent, and both parents absent. However, prior studies indicated parental gender differences. That is, father’s absence and mother’s absence may impact the male and female adolescent’s mental health differently [2]. In future research, both parent’s gender and adolescent’s gender should be considered to explore gender specific differences and similarities. Finally, the data for this study came from the less developed, multi-ethnic regions in southwest China. As such, care must be taken when generalizing the study findings to adolescents residing in other regions of China.

## Conclusions

The PYD theoretical framework prioritizes the development of external and internal assets during adolescents’ growth and underscores the positive impacts of development assets on adolescents’ mental health. Active participation in organized physical activity, as a protective factor, can help adolescents develop and improve their psychosocial skills and development assets in the school context, which can, in turn, improve their subjective well-being. This holds true especially for left-behind adolescents. The findings of this study provide new ideas for related researchers to further explore the positive effects of PYD-centered physical activity interventions on adolescents in future studies to improve the subjective well-being of adolescents in less developed, multi-ethnic regions such as southwest China.

## Supplementary Information

**Additional file 1.** 

**Additional file 2.**

## Data Availability

The datasets generated and analyzed in the current study aren't publicly available due to restrictions on anonymous ethical approval. However, the above information can be obtained from the corresponding authors upon reasonable request.
