# Supplementary material for: The association between physical activity and subjective well-being among adolescents in southwest China by parental absence: a moderated mediation model
Source: BMC Psychiatry. 2023 Jul 10;23:493. doi: 10.1186/s12888-023-04982-8 (PMC10332099; doi:10.1186/s12888-023-04982-8)
Supplement: Supplementary file 1 — Additional file 1. [file 12888_2023_4982_MOESM1_ESM.pdf]

Appendix 1

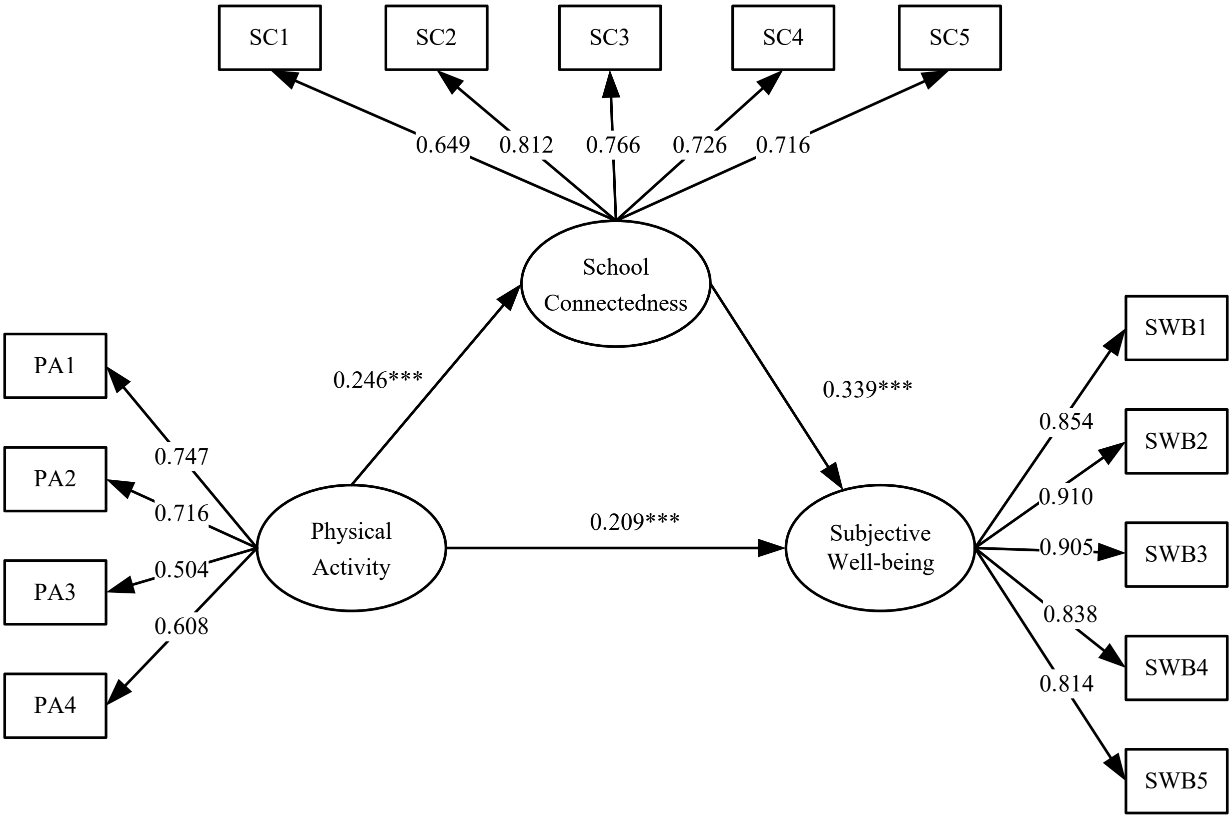

Appendix Fig. 1 Standardized SEM coefficients (*N*=3143) for the association between physical activity and subjective well-being among adolescents in southwest China: the mediating role of school connectedness.

Notes: PA1-PA4 represent the 4 items of the physical activity scale, SC1-SC5 represent the 5 items of the school connectedness scale, and SWB1-SWB5 represent the 5 items of the WHO-5 scale. And the pathways from the control variables (i.e., gender, age, and ethnicity) to subjective well-being are omitted. ****P*<0.001 (two-tailed).

Appendix Table 1 Multicollinearity test

Variable	Unstandardized coefficient		Standardized coefficient	*t*	*P*	Co-linear statistics	
	B	*SE*	*β*			Tolerance	VIF
**Age**	-0.010	0.009	-0.023	-1.197	0.232	0.877	1.141
**Gender**	-0.009	0.027	-0.006	-0.320	0.749	0.976	1.024
**Ethnicity**	-0.134	0.020	-0.124	-6.781	0.000	0.938	1.066
**Physical activity**	0.028	0.006	0.090	4.740	0.000	0.868	1.152
**School connectedness**	-0.005	0.004	-0.026	-1.313	0.189	0.788	1.270
**Subjective well-being**	0.001	0.002	0.005	0.239	0.811	0.644	1.553
**Resilience**	-0.002	0.001	-0.050	-2.342	0.019	0.694	1.442

Notes: Dependent variable is parental absence.

Appendix Table 2 Path coefficients of the mediation model

Path	Unstandardized coefficient	Standardized coefficient	*SE*	*t*	*P*
**Physical activity → School connectedness**	0.428	0.246	0.040	10.634	<0.001
**School connectedness → Subjective well-being**	0.696	0.339	0.042	16.753	<0.001
**Physical activity → Subjective well-being**	0.744	0.209	0.073	10.174	<0.001
**Gender → Subjective well-being**	-0.119	-0.044	0.045	-2.663	0.008
**Age → Subjective well-being**	-0.132	-0.163	0.013	-9.864	<0.001
**Ethnicity → Subjective well-being**	0.280	0.145	0.032	8.779	<0.001

Appendix Table 3a Multi-group comparison of adaptation

Model	CMIN	*DF*	*P*	CMIN/*DF*
**Unconstrained**	2052.108	660	<0.001	3.109
**Measurement weights**	2082.752	690	<0.001	3.018
**Structural weights**	2118.726	710	<0.001	2.984
**Structural covariances**	2435.191	728	<0.001	3.345
**Structural residuals**	2449.502	732	<0.001	3.346
**Measurement residuals**	2552.015	772	<0.001	3.306

Appendix Table 3b Multi-group comparison of adaptation

Model	NFI	RFI	IFI	TLI	CFI	GFI	AGFI	PGFI	RMSEA	AIC	ECVI
**Unconstrained**	0.945	0.937	0.962	0.956	0.962	0.946	0.932	0.754	0.026	2388.108	0.761
**Measurement weights**	0.944	0.939	0.962	0.958	0.962	0.945	0.934	0.788	0.025	2358.752	0.751
**Structural weights**	0.943	0.939	0.962	0.959	0.962	0.944	0.935	0.810	0.025	2354.726	0.750
**Structural covariances**	0.935	0.932	0.953	0.951	0.953	0.938	0.929	0.824	0.027	2635.191	0.839
**Structural residuals**	0.934	0.932	0.953	0.951	0.953	0.937	0.929	0.829	0.027	2641.502	0.841
**Measurement residuals**	0.932	0.933	0.951	0.952	0.951	0.935	0.931	0.872	0.027	2664.015	0.848

Appendix Table 4 Invariance test

Model	delta-CMIN	delta-*DF*	*P*	delta-NFI	delta-RFI	delta-IFI	delta-TLI	delta-CFI	delta-GFI	delta-AGFI	delta-PGFI
**Measurement weights**	30.644	30	0.433	-0.001	0.002	0.000	0.002	0.000	-0.001	0.002	0.034
**Structural weights**	35.974	20	0.015	-0.001	0.000	0.000	0.001	0.000	-0.001	0.001	0.022
**Structural covariances**	316.465	18	<0.001	-0.008	-0.007	-0.009	-0.008	-0.009	-0.006	-0.006	0.014
**Structural residuals**	14.311	4	0.006	-0.001	0.000	0.000	0.000	0.000	-0.001	0.000	0.005
**Measurement residuals**	102.513	40	<0.001	-0.002	0.001	-0.002	0.001	-0.002	-0.002	0.002	0.043
