# Supplementary material for: The association between physical activity and subjective well-being among adolescents in southwest China by parental absence: a moderated mediation model
Source: BMC Psychiatry. 2023 Jul 10;23:493. doi: 10.1186/s12888-023-04982-8 (PMC10332099; doi:10.1186/s12888-023-04982-8)
Supplement: Supplementary file 2 — Additional file 2. [file 12888_2023_4982_MOESM2_ESM.pdf]

Appendix 2 The Connor-Davidson resilience scale (CD-RISC)

Item	0=not true at all	1=rarely true	2=sometimes true	3=often true	4=true nearly all of the time
1. Able to adapt to change					
2. Close and secure relationships					
3. Sometimes fate or God can help					
4. Can deal with whatever comes					
5. Past success gives confidence for new challenge					
6. See the humorous side of things					
7. Coping with stress strengthens					
8. Tend to bounce back after illness or hardship					
9. Things happen for a reason					
10. Best effort no matter what					
11. You can achieve your goals					
12. When things look hopeless, I don't give up					
13. Know where to turn for help					
14. Under pressure, focus and think clearly					
15. Prefer to take the lead in problem solving					
16. Not easily discouraged by failure					
17. Think of self as strong person					
18. Make unpopular or difficult decisions					
19. Can handle unpleasant feelings					
20. Have to act on a hunch					
21. Strong sense of purpose					
22. In control of your life					
23. I like challenges					
24. You work to attain your goals					
25. Pride in your achievements					

Notes: Items 11, 12, 13, 14, 15, 16, 17, 18, 19, 20, 21, 22, and 23 are the tenacity subscale; items 1, 5, 7, 8, 9, 10, 24, and 25 are the strength subscale; and items 2, 3, 4, and 6 are the optimism subscale.
